# COVID-19-Associated Pulmonary Aspergillosis Isolates Are Genomically Diverse but Similar to Each Other in Their Responses to Infection-Relevant Stresses

**DOI:** 10.1128/spectrum.05128-22

**Published:** 2023-03-22

**Authors:** Matthew E. Mead, Patrícia Alves de Castro, Jacob L. Steenwyk, Jean-Pierre Gangneux, Martin Hoenigl, Juergen Prattes, Riina Rautemaa-Richardson, Hélène Guegan, Caroline B. Moore, Cornelia Lass-Flörl, Florian Reizine, Clara Valero, Norman Van Rhijn, Michael J. Bromley, Antonis Rokas, Gustavo H. Goldman, Sara Gago, on behalf of the ECMM CAPA Study Group

**Affiliations:** a Department of Biological Sciences and Evolutionary Studies Initiative, Vanderbilt University, Nashville, Tennessee, USA; b Faculdade de Ciências Farmacêuticas de Ribeirão Preto, Universidade de São Paulo, Ribeirão Preto, São Paulo, Brazil; c University of Rennes, CHU Rennes, Inserm, EHESP, IRSET (Institut de recherche en santé, environnement et travail), Rennes, France; d Division of Infectious Diseases, Medical University of Graz, Graz, Austria; e Biotech Med, Graz, Austria; f Mycology Reference Centre Manchester and Department of Infectious Diseases, Manchester University, Manchester University NHS Foundation Trust, Wythenshawe Hospital, Manchester, United Kingdom; g Division of Evolution, Infection and Genomics, School of Biological Sciences, Faculty of Biology, Medicine and Health, The University of Manchester, Manchester, United Kingdom; h European Excellence Center for Medical Mycology (ECMM), Institute of Hygiene and Medical Microbiology, Medical University of Innsbruck, Austria; i Medical Intensive Care Unit, Rennes University Hospital, Rennes, France; j Manchester Fungal Infection Group, School of Biological Sciences, Faculty of Biology, Medicine and Health, The University of Manchester, Manchester, United Kingdom; University of Guelph

**Keywords:** strain heterogeneity, *Aspergillus fumigatus*, fungal pathogen, coinfection, SARS-CoV-2

## Abstract

Secondary infections caused by the pulmonary fungal pathogen Aspergillus fumigatus are a significant cause of mortality in patients with severe coronavirus disease 19 (COVID-19). Even though epithelial cell damage and aberrant cytokine responses have been linked to susceptibility to COVID-19-associated pulmonary aspergillosis (CAPA), little is known about the mechanisms underpinning copathogenicity. Here, we analyzed the genomes of 11 A. fumigatus isolates from patients with CAPA in three centers from different European countries. CAPA isolates did not cluster based on geographic origin in a genome-scale phylogeny of representative A. fumigatus isolates. Phenotypically, CAPA isolates were more similar to the A. fumigatus A1160 reference strain than to the Af293 strain when grown in infection-relevant stresses, except for interactions with human immune cells wherein macrophage responses were similar to those induced by the Af293 reference strain. Collectively, our data indicate that CAPA isolates are genomically diverse but are more similar to each other in their responses to infection-relevant stresses. A larger number of isolates from CAPA patients should be studied to better understand the molecular epidemiology of CAPA and to identify genetic drivers of copathogenicity and antifungal resistance in patients with COVID-19.

**IMPORTANCE** Coronavirus disease 2019 (COVID-19)-associated pulmonary aspergillosis (CAPA) has been globally reported as a life-threatening complication in some patients with severe COVID-19. Most of these infections are caused by the environmental mold Aspergillus fumigatus, which ranks third in the fungal pathogen priority list of the WHO. However, little is known about the molecular epidemiology of Aspergillus fumigatus CAPA strains. Here, we analyzed the genomes of 11 A. fumigatus isolates from patients with CAPA in three centers from different European countries, and carried out phenotypic analyses with a view to understanding the pathophysiology of the disease. Our data indicate that A. fumigatus CAPA isolates are genomically diverse but are more similar to each other in their responses to infection-relevant stresses.

## INTRODUCTION

Lung coinfections and super infections caused by either bacteria or fungi are frequent and increase mortality in patients with severe COVID-19 (1, 2). Among fungal species known to cause secondary infections in patients already infected with severe acute respiratory syndrome coronavirus 2 (SARS-CoV2), Aspergillus species can give rise to COVID-19-associated pulmonary aspergillosis (CAPA) in about 15.1% of ICU-admitted COVID-19 patients (3). However, the incidence of CAPA varies across medical centers and has been reported to range between 0.7 and 34.4%. Nevertheless, in a retrospective study of the literature (4), it was reported that 52.5% of patients with CAPA died early after the diagnosis of the disease (<6 weeks after CAPA diagnosis), and 33.0% of these deaths were attributed to aspergillosis. Therefore, we need to improve our understanding of the molecular epidemiology of Aspergillus fumigatus CAPA strains with a view to better understand the disease and its impact on human health (5).

Infections due to A. fumigatus are the most common cause of CAPA, but other Aspergillus species have been recently found in the clinic (3, 4). Attempts to understand why A. fumigatus is the most frequent cause of aspergillosis have been carried out for decades (6). Several of these studies have focused on understanding traits related to its virulence in susceptible hosts (7 to 12). Several studies have shown A. fumigatus phenotypic heterogeneity in infection-relevant traits, and this has been linked to differences in virulence (13 to 15). Moreover, phenotypic heterogeneity is largely attributed to genomic heterogeneity between A. fumigatus isolates (16 to 18), since approximately 16 to 42% of the genome of an A. fumigatus isolate is variable (16, 19).

Different mechanisms generate diversity and can facilitate adaptation to specific niche environments in A. fumigatus. For example, the generation of genetic variation in patients with chronic pulmonary infections has been linked to parasexual recombination (20) and the emergence of nonsynonymous mutations (21). Less is known regarding the heterogeneity of the phenotypes and genomes of A. fumigatus CAPA isolates, as the disease is relatively new. To elucidate whether genomic- and pathogenicity-related characteristics in CAPA isolates are similar to non-CAPA, but clinically relevant, isolates, we previously analyzed the genomic, chemical, and phenotypic heterogeneity of four CAPA isolates from Germany (5). Surprisingly, we found that the four CAPA isolates were more closely related to each other than to other A. fumigatus isolates and displayed only some degree of phenotypic heterogeneity. Aiming to better understand whether this lack of genomic diversity holds true across CAPA isolates, we built upon our previous study and performed genomic and phenotypic traits analyses of 11 additional A. fumigatus CAPA isolates from three European centers based at Graz (Austria), Manchester (UK), and Rennes (France). We observed that A. fumigatus CAPA isolates are genomically diverse but are more similar to each other in their responses to infection-relevant stresses. We conclude that A. fumigatus CAPA isolates likely span the genomic and phenotypic diversity of A. fumigatus.

## RESULTS AND DISCUSSION

### New CAPA isolates represent diverse lineages of the A. fumigatus phylogeny.

To determine the evolutionary relationships between the 11 newly identified and sequenced CAPA isolates, four previously analyzed CAPA isolates, 55 A. fumigatus strains, and three outgroup taxa (two strains of A. fischeri and one of A. oerlinghausenensis, the closest known relatives of A. fumigatus [22, 23]) were used to infer the phylogeny of these strains (Fig. S1, Table S1 at https://doi.org/10.6084/m9.figshare.21688172). Our tree showed that the 11 new CAPA isolates belonged to A. fumigatus; however, these 11 new isolates were more diverse than the four previously sequenced CAPA isolates. The four previously sequenced CAPA isolates all originated from Germany and were very closely related to each other and to the strains A1163 and Af293 (5). In contrast, none of the new CAPA isolates are closely related to A1163 and Af293 or the previously sequenced CAPA isolates; instead, these isolates span the A. fumigatus phylogeny.

**FIG 1:**
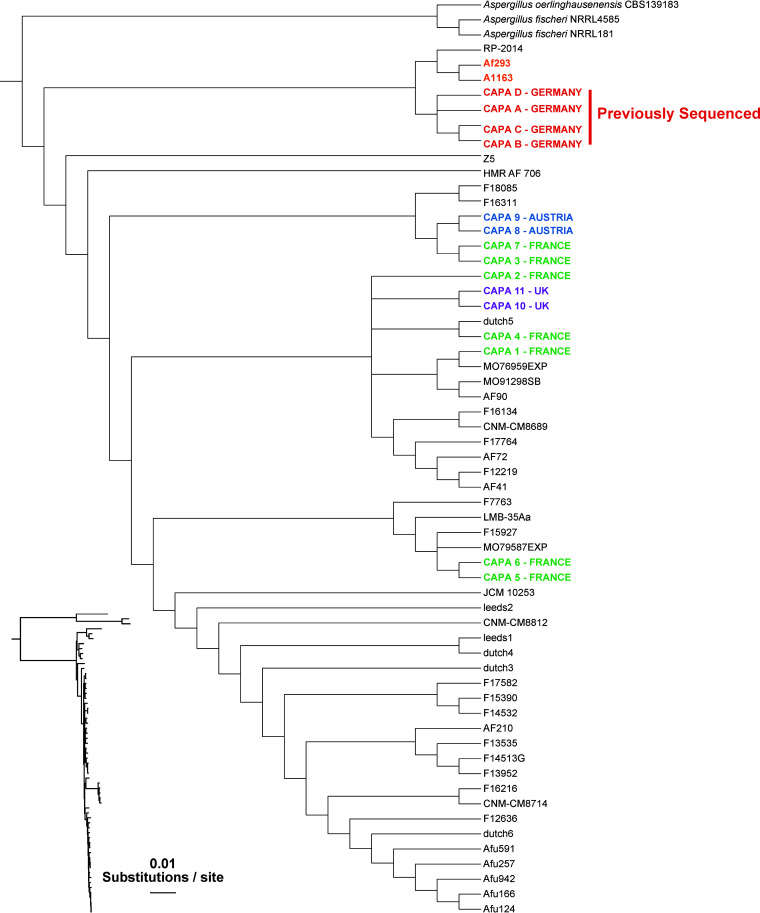
Phylogeny of 55 A. fumigatus isolates and 3 outgroup taxa reveals that the 11 new CAPA isolates span the genomic diversity of the species. We used 4,515 single-copy orthologs from a total of 61 taxa (11 new CAPA isolates, three non-A. fumigatus outgroup strains, 43 A. fumigatus isolates that span the diversity of the species, and 4 previously analyzed CAPA isolates) as input to construct a maximum likelihood tree. A slight geographic clustering of CAPA isolates was observed, but isolates from different locales were more diverse than a previous set of four CAPA isolates (all of which were isolated from the same hospital in Germany).

Two A. fumigatus CAPA isolates from Austria and two CAPA isolates from a patient from the United Kingdom were most closely related to each other, respectively. The original four German isolates came from two different centers in Cologne, Germany (5). While the closest relatives of most CAPA isolate clades are clades of non-CAPA isolates, that is not the case for CAPA-8/9 (from Austria) and CAPA-3/7 (from France), which are most closely related to each other (see Fig. 1). These results suggest that there are likely to be few or no genomic traits that are uniquely shared only by CAPA isolates from the same hospital. Interestingly, this finding is in disagreement with our previously published data showing that CAPA isolates from the same geographic area are closely related, thus suggesting a possible common source of infection (5).

### Strain heterogenicity of CAPA isolates in virulence-related culture conditions and antifungal drug susceptibility.

**FIG 2:**
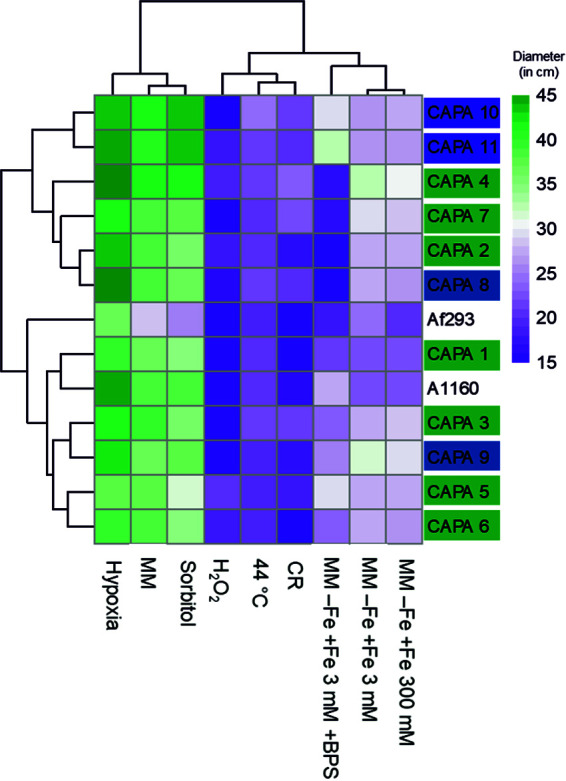
A. fumigatus radial growth in infection-relevant culture media. Data are represented as mean of colony diameter (mm) of A. fumigatus CAPA strains and controls. Clustering of isolates was carried out according to diameter size. Isolate name is color-coded according to geographical origin.

A. fumigatus isolates from CAPA patients displayed strain-dependent variation in growth phenotypes compared to the reference strains Af293 and A1160 (Fig. 2; Fig. S1 at https://doi.org/10.6084/m9.figshare.21688172). In general, most CAPA isolates displayed phenotypes similar to the reference strain A1160 when grown under hypoxia, osmotic stress, high temperature (44°C), or low and high concentrations of iron. For three of the CAPA isolates, radial growth in the presence of cell-wall stress (CAPA 6), oxidative stress (CAPA 7 and 9), and/or iron starvation stress (CAPA 6 and 7) was significantly reduced compared to both Af293 and A1160 (two-way ANOVA with Dunnett’s *post hoc* test, *P* < 0.05). Reduced sensitivity to cell-wall-damaging agents was not detected in the 11 CAPA isolates included in this study. However, we used Congo red as a stressor rather than calcofluor white, which was used in Steenwyk et al. (5). All CAPA isolates grow similarly to A. fumigatus reference strains when cultured in solid minimal media (MM; without any stress). Additionally, there were no statistically significant differences among CAPA isolates for any of the *in vitro* phenotypic conditions assayed in this study.

All CAPA isolates were susceptible to amphotericin B, isavuconazole, itraconazole, and voriconazole. The MECs obtained for the echinocandins are comparable to other Aspergillus species tested (6). Posaconazole MICs ranged from 0.06 to 0.25 mg/L and were converted to the highest concentration detected. The resistant CBP is defined as >0.5 mg/L, and an MIC of 0.25 mg/L was recently designated an area of technical uncertainty (24) potentially displaying wild-type and non-wild-type populations. In such cases, testing of itraconazole is recommended, and if it is susceptible, the strain is designated to be susceptible against posaconazole (25). In addition, we tested the strains twice, and the MICs obtained never exceeded 0.25 mg/L. All quality control strains were within the tested ranges. (Table 1).

**TABLE 1:** Susceptibility profile of the 11 CAPA isolates included in this study at 48 h^*a*^

Isolate	MIC and MEC (mg/L)						
	AMB	CSP	MCF	PCZ	VCZ	IVZ	ITR
1	0.5	0.125	0.032	0.25	1	0.5	0.5
2	0.5	0.125	0.032	0.25	1	1	0.5
3	0.5	0.125	0.032	0.25	1	1	0.5
4	0.5	0.125	0.032	0.25	0.5	1	1
5	0.5	0.125	0.032	0.25	1	1	0.5
6	1	0.125	0.032	0.25	1	1	1
7	1	0.25	0.032	0.25	0.5	0.5	0.5
8	0.5	0.25	0.032	0.25	1	1	0.5
9	0.5	0.25	0.032	0.25	1	1	1
10	0.5	0.25	0.032	0.25	1	1	0.5
11	0.5	0.25	0.032	0.25	1	1	0.5

a AMB, amphotericin B; CSP, caspofungin; MCF, micafungin; PCZ, posaconazole; VCZ, voriconazole; IVZ, isavuconazole; ITR, itraconazole. Isolates were tested twice. MICs given were converted to the highest concentration detected.

### CAPA isolates are more efficiently killed by macrophages than reference strain A1160.

**FIG 3:**
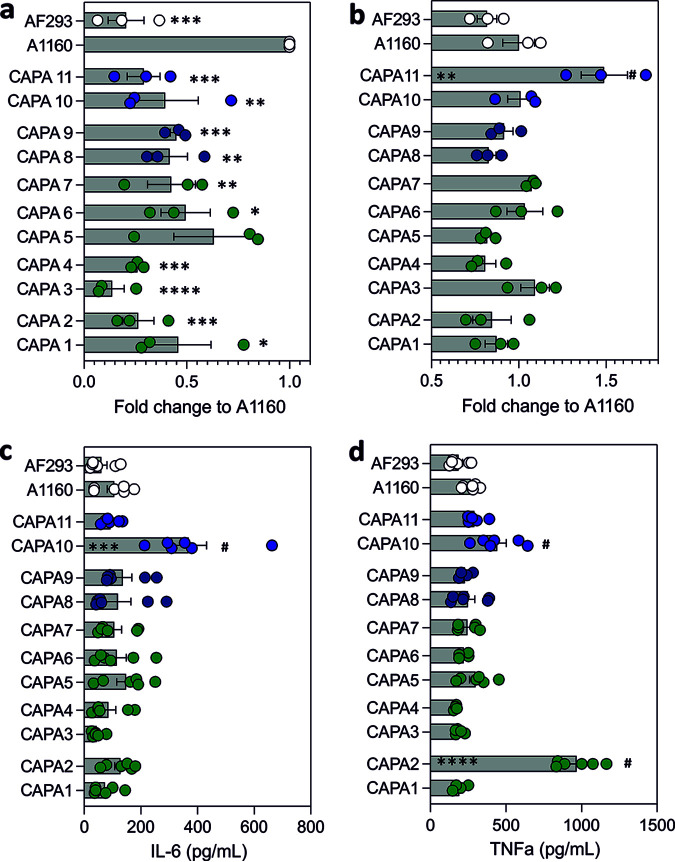
CAPA isolates exhibit *in vitro* RAW 263.7 macrophage responses similar to Af293. (a) Fold change killing of A. fumigatus CAPA isolates and reference strains Af293 and A1160 by RAW 263.7 macrophages at 6 h postinfection. (b) LDH release of macrophages challenged with 11 CAPA isolates and reference strains Af293 and A1160 at 24 h postinfection. IL-6 (c) and TNFa (d) release by RAW 263.7 macrophages at 9 h postinfection with CAPA strains and controls. Data represent mean and standard deviation of a minimum of three biological and technical replicates. Neat controls were subtracted from test samples and used for background corrections. *, *P* < 0.05; **, *P* < 0.01; ***, *P* < 0.001; ***, *P* < 0.0001 compared to A1160. #, *P* < 0.05 compared to other CAPA strains.

Macrophage killing of A. fumigatus conidia is one of the main mechanisms of antifungal defense during infection. Pulmonary macrophages in COVID-19 have been described to be hyperactivated, thus favoring tissue damage at the site of infection (26). The efficiency of macrophages to kill A. fumigatus CAPA isolates and two other reference isolates was comparatively analyzed at 6 h postinfection. CAPA isolates were less susceptible to macrophage killing compared to reference strain A1160 but exhibited killing rates on par with those of reference strain Af293. There were no differences in susceptibility to macrophage killing among CAPA strains (Fig. 3a; *P* < 0.05). *In vivo* studies using immunosuppressed mouse models of infection and neutrophil-depleted zebra fish larvae have shown that A. fumigatus strain CEA10, for which A1160 is a derivative, is more virulent than Af293 (14, 27, 28). However, our data indicate decreased killing of A. fumigatus Af293 *in vitro* compared to the CEA10 derivative A1160. Moreover, it has been recently reported that A. fumigatus Af293 might be more pathogenic in immunocompetent hosts than CEA10 (29). Differences in A. fumigatus killing were not correlated with differential cytokine profiles at 9 h postinfection (Fig. 3b and c). Only A. fumigatus CAPA isolates 2 and 10 showed increased secretion of IL-6 and/or TNF-α compared to any of the control and CAPA strains at 9 h postinfection. Differences in the capacity of macrophages to kill CAPA isolates compared to A. fumigatus controls were not linked to decreased cell cytotoxicity as measured by lactate dehydrogenase (LDH) release (Fig. 3d). Altogether, these data indicate that the CAPA isolates included in this study have generally similar pathogenicity profiles, during infection with macrophages, to the reference strain Af293.

In this study, we found A. fumigatus isolates from patients with CAPA are genetically heterogenous but phenotypically similar. Increasing the number of available CAPA genomes since our previous study has allowed us to observe that CAPA isolates represent diverse lineages of the A. fumigatus phylogeny and the concomitant absence of geographical clusters, which contrasts with our earlier findings based on an analysis of four CAPA isolates from two different hospitals in Cologne, Germany (5). The 11 new CAPA isolates have similar phenotypic profiles to A. fumigatus A1160 when tested for sensitivity to stressors in culture-relevant conditions, while macrophage resistance phenotypes were more similar to Af293. However, this may be an artifact due to the relatively small number of isolates included in the study. Similarly, our previous study indicated that CAPA isolate secondary metabolite profiles were more similar to A. fumigatus Af293 than to A1160 (5). Recent work suggests that differences in virulence for A. fumigatus A1160 (or the parental strain CEA10) and Af293 might be determined by the experimental system used (30). However, it has been reported that A. fumigatus Af293 but not A1160 triggers SARS-CoV2 replication within airway epithelial cells (31). Further studies including a higher number of CAPA isolates are required to confirm what specific fungal genetic factors are regulators of fungal pathogenesis in these patients. In-host microevolution of A. fumigatus strains during chronic infections has been reported in the literature (20, 21). Even though this has only been reported in long-term infections, a previous study attempted to determine whether specific single nucleotide polymorphisms (SNPs) or copy number variants in genetic determinants of virulence and biosynthetic gene clusters could explain A. fumigatus CAPA genomic heterogeneity (5). An early stop codon in *pptA* was found, but this did not correlate with reduced production of secondary metabolites in CAPA isolates.

A limitation of this study is the small number of isolates included and the absence of any A. fumigatus isolate resistant to antifungal drugs. However, other studies have reported the recovery of azole-resistant isolates in patients with CAPA, thus supporting the clinical value of antifungal susceptibility testing in these patients (32 to 34). In addition, point mutations in the azole target enzyme in A. fumigatus 14-α sterol demethylase (*Cyp51A*) have been recently described in patients with CAPA; these include the TR34/L98H alteration in the promoter region and G54RF46Y, M172V, and E427K (33). Therefore, a higher number of resistant CAPA isolates should be sequenced to understand whether other mutations in this or other genes might underpin antifungal resistance in CAPA. Even though we have not collected environmental strains from the hospitals where the isolates were obtained, isolates from different patients within the same center were so diverse that it is unlikely that the infections were hospital-acquired as previously reported in other CAPA cohorts (33, 35). Overall, continued genome sequencing and phenotypic characterization of additional CAPA isolates, such as those we report here, may facilitate identifying mutations that impact infection-relevant traits or are responsible for antifungal resistance. These genome-sequencing approaches will also aid in investigating the possible emergence of A. fumigatus genotypes that might be causing infection in a particular setting.

## MATERIALS AND METHODS

### Patient information and ethics approval.

Aspergillus fumigatus isolates (*n* = 11) were obtained as part of the multinational CAPA observational study of the European Confederation of Medical Mycology (36). Each participating study center (Manchester, Graz, and Rennes) was responsible for obtaining local institutional review board and/or local ethics policy approval. Institutional review board approval numbers are as follows: Medical University of Graz EC number 32-296 ex 19/20; at the University of Manchester, data acquisition was conducted as a retrospective audit, which does not require local ethics but was approved by the hospital's audit committee; at Rennes University Hospital, this protocol was approved by the local ethics committee (approval number 20.56). The study has been performed in accordance with the ethical standards laid down in the 1964 Declaration of Helsinki and its later amendments. Patient demographics are summarized in Table 2. Each strain was recovered from an individual patient except for strains 10 and 11 (Manchester, UK), where the two A. fumigatus isolates were from the same patient. None of the patients were on antifungal prophylaxis before the strain was recovered from culture except for patient 9. All patients were on azole therapy after diagnosis of the infection.

**TABLE 2:** Patient and isolate information^*a*^

Isolate name	City/country of origin	Underlying condition	Age	Gender	Sample origin	Antifungal prophylaxis	Antifungal treatment
CAPA 1	Rennes, France	Chronic myeloid leukemia, ARDS	79	Female	BAL + tracheal aspiration	No	VCZ
CAPA 2	Rennes, France	Chronic myelo-monocytic leukemia, ARDS	78	Female	Tracheal aspiration	No	VCZ
CAPA 3	Rennes, France	ARDS	75	Male	Tracheal aspiration	No	VCZ
CAPA 4	Rennes, France	ARDS	58	Male	Tracheal aspiration	No	VCZ
CAPA 5	Rennes, France	ARDS	78	Female	Tracheal aspiration	No	VCZ
CAPA 6	Rennes, France	ARDS, obesity, hypertension	71	Male	Tracheal aspiration	No	VCZ
CAPA 7	Rennes, France	ARDS, hypertension	73	Male	Tracheal aspiration	No	VCZ
CAPA 8	Graz, Austria	ARDS	65	Male	Tracheal aspiration	No	IVZ
CAPA 9	Graz, Austria	ARDS	60	Male	BAL	Yes (PSZ)	IVZ
CAPA 10	Manchester, UK	ECMO	41	Male	BAL	No	VCZ
CAPA 11	Manchester, UK	ECMO	41	Male	BAL	No	VCZ

a ARDS, acute respiratory distress syndrome; CAPA, COVID-19 associated pulmonary aspergillosis; ECMO, extracorporeal membrane oxygenation; BAL, bronchoalveolar lavage; PSZ, posaconazole; VCZ, voriconazole; IVZ, isavuconazole. Note that CAPA 10 and 11 were isolated from the same patient.

### DNA extraction and sequencing.

All 11 A. fumigatus CAPA isolates (Table 2) were grown from 1 × 10^7^ asexual spores (conidia) in MM (1% [wt/vol] glucose, nitrate salts, trace elements, pH 6.5) (37, 38) for 20 h at 37°C. After mechanical disruption of the mycelia, genomic DNA extraction was performed in phenol:chloroform (1:1). DNA quantity and quality were assessed using a NanoDrop 2000 spectrophotometer (Thermo Scientific). The DNA purity ranged from 1.8 to 2.0 for OD260/280 and 2.0 to 2.2 for OD260/230.

Library preparation and sequencing was carried out by Vanderbilt Technologies for Advanced Genomics (VANTAGE). Libraries were prepared using the NEBNext Ultra II DNA Library Prep kit. Sequencing of the libraries was carried out on an Illumina NovaSeq 6000 to produce paired-end, 150-bp reads.

### *De novo* genome assembly, annotation, and quality determination.

To obtain high-quality and adapter-free reads, raw reads were trimmed with Trimmomatic version 0.39 (39) using the parameters “2:30:10 LEADING:3 TRAILING:3 SLIDINGWINDOW:4:15 MINLEN:36.” On average, 36 million read pairs passed trimming. Trimmed reads were then assembled with SPAdes version 3.15.2 (40) using the parameters “–isolate” and “–cov-cutoff auto.” Genome statistics were calculated with BioKIT version 0.0.4 (41).

To identify putative protein-coding genes, Augustus version 3.3.2 (42) was used to annotate the newly assembled CAPA genomes. The Aspergillus fumigatus annotation that is packaged with the software was used as a training data set. Completeness and fragmentation of the genomes were determined with version 4.0.4 of BUSCO (43) using the default Eurotiales database. All quality metrics for the genome assemblies and annotations of the new CAPA isolates were comparable to values for reference strains Af293 and A1163 (7, 8, 44).

### Phylogenomic tree inference.

To determine the general taxonomy of the 11 new CAPA isolates, we built a single gene tree of the *tef1* homologs from the new CAPA strains and the 100 genes most similar to the Tef1 ortholog present in A. fumigatus strain Af293 (XM_745295.2). Homologs of A. fumigatus Tef1 were identified in both the CAPA isolates and the NCBI nucleotide collection (nr/nt database) with blastn version 2.8.1 (45) using default parameters. The 112 *tef1* sequences (11 CAPA + 100 NCBI + 1 A. fumigatus Af293) were aligned with MAFFT version 7.402 (46, 47) and the following parameters: -op 1.0 -maxiterate 1000 -retree 1 -genafpair. The resulting alignment was trimmed with version 1.2.0 of ClipKIT (22), and a tree was made from the trimmed alignment using version 1.6.12 of IQ-TREE with the model finder parameter and 5,000 ultrafast bootstraps (48). A cladogram of the tree was visualized with iTOL version 5 (49).

To infer the phylogenetic relationships between the 11 new CAPA isolates and other A. fumigatus strains, a modified version of a previously published pipeline was employed (5). Specifically, genomes from 50 taxa (three non-A. fumigatus outgroup strains, 43 A. fumigatus isolates that span the diversity of the species, and four previously analyzed CAPA isolates) were obtained from our previous study (https://doi.org/10.6084/m9.figshare.13118549) (5) and compared to the genomes of the 11 new CAPA isolates.

To discover suitable loci for phylogenetic reconstruction, 4,525 single-copy orthologs identified among the 50 previously analyzed genomes were obtained. A Hidden Markov Model (HMM) was made for each orthologous group of genes using hmmbuild within HMMER version 3.2.1 (hmmer.org). These 4,525 HMMs were used as input in orthofisher version 1.0.3 (50) to identify the copies of the orthologs in the genomes of the 11 new CAPA isolates with the parameter “-b 0.95.” Ten of the orthologs were found to vary in their copy number across the 11 new genomes and were not used in subsequent analyses. Protein sequences from the 4,515 single-copy orthologs from all 61 taxa were combined into 4,515 FASTA files for further analysis.

To align the 4,515 single-copy orthologs, MAFFT version 7.402 (46, 47) was used along with the parameters “-bl 62 -op 1.0 -maxiterate 1000 -retree 1 -genafpair” (41). The 4,515 alignments were trimmed with version 1.2.0 of ClipKIT (22) and then combined into a supermatrix with PhyKIT version 1.5.0 (51). The resulting supermatrix contained 2,361,569 amino acid sites and was analyzed using IQ-TREE version 1.6.12 (48, 52) and parameters “-bb 5000 -m TEST -nbest 10 –runs 5 -safe” to produce a maximum likelihood tree. Note that these parameters included using 5,000 ultrafast bootstrap support approximations (53). The tree was visualized with iTOL version 5 (49).

### Antifungal susceptibility testing.

To evaluate the susceptibility of various antifungal drugs, antifungal susceptibility testing of the CAPA isolates was performed using the EUCAST (European Committee for Antimicrobial Susceptibility Testing) reference microdilution method version 9.3.2 (54). Susceptibility to amphotericin B, isavuconazole, voriconazole, posaconazole, itraconazole, caspofungin, and micafungin was tested at 48 h. All antifungals were purchased from Sigma-Aldrich (Vienna, Austria). Candida parapsilosis ATCC 22019 and Aspergillus fumigatus ATCC 204305 were used as quality-control strains. *In vitro* tests were performed in duplicate. A categorization according to epidemiological cutoffs (ECOFFs) and clinical breakpoints (CBPs) was applied (24). Susceptible is <1 mg/L for amphotericin B, isavuconazole, and voriconazole; for the echinocandins, neither ECOFFS nor CBP are available. MICs falling within ± 2 dilutions (due to double testing) were converted to the highest concentration detected.

### Growth assays.

Aspergillus fumigatus radial growth from CAPA isolates and the reference strains Af293 and A1160 was comparatively analyzed on either solid minimal media (MM) or MM supplemented with different concentrations of stressor agents (sorbitol [1 M], Congo red [10 mg/mL], or hydrogen peroxide [1.5 mM]) at 37°C. For the different iron availability conditions, iron was omitted from the trace element solution (38) and supplemented at various concentrations (3 μM for iron depletion or 300 μM for iron excess). The ferreous iron chelator bathophenanthroline disulfonic acid (BPS) was used at 200 μM to increase iron starvation in solid media as described in Gsaller et al. (55). Plates were inoculated with 10^4^ spores per strain, and growth was then measured after 72 h. Radial growths were expressed as ratios, dividing colony radial diameter of growth in the stress condition by colony radial diameter in the control (no stress) condition. The capacity to grow under hypoxia (5% CO_2_ 1% O_2_) and at 44°C was also evaluated. Experiments were done using two or more biological and technical replicates. Statistical comparisons of growth rate of the CAPA isolates versus reference strains Af293 and A1160 were done using two-way ANOVA with Dunnett’s *post hoc* test (GraphPad Prism v9, La Jolla, CA). Statistical comparisons among CAPA isolates were carried out using two-way ANOVA with Turkey’s *post hoc* test (GraphPad Prism v9, La Jolla, CA).

### Pathogenicity assays.

To investigate differences in A. fumigatus killing by macrophages, 10^6^ RAW 264.7 cells were seeded in 6-well plates and incubated for 24 h. RAW 264.7 murine macrophages (ATCC TIB-71) were maintained at 37°C, 5% CO_2_ in Dulbecco’s modified Eagle’s medium (DMEM) supplemented with 10% fetal bovine serum (FBS) and a 1% penicillin–streptomycin solution, all from Merck (Darmstadt, Germany). Macrophages were used under passage 20. Cells were then challenged with 10^6^ spores of each of the isolates and incubated for 6 h. Cells were then lysed in water and plated in Sabouraud agar plates. CFU were enumerated after 24 h of incubation at 37°C. To correct for strain heterogenicity, the number of CFU for a particular isolate in confrontation experiments with macrophages was divided by the number of CFU for that isolate in the absence of macrophages. Experiments were done using three or more biological replicates and technical duplicates. Statistical comparisons of macrophage killing of A. fumigatus CAPA isolates and the reference strains A1160 and Af293 were done by one-way ANOVA with Dunnett’s *post hoc* tests while Tukey’s *post hoc* test was used for comparisons among CAPA isolates (GraphPad Prism version 9, La Jolla, CA).

It has been previously described that A. fumigatus germination is critical to induce cytokine responses and cytotoxicity of host cells during infection. To investigate whether A. fumigatus CAPA isolates were able to induce host-cell damage and activate macrophage responses in a different manner than the reference strains Af293 and A1160, 10^6^ RAW 264.7 macrophages were seeded in 24-well plates and challenged with 10^6^ spores (56) for 9 and 24 h. LDH release was measured using the Cyto Tox 96 Non-Radioactive Cytotoxicity Assay (Promega, Madison, WI, USA) according to manufacturer’s instructions. The concentrations of IL-6 and TNF-α in cell culture supernatants was measured by using the Mouse IL-6 and TNF-α DuoSet ELISA (R&D Systems, Minneapolis, MN, USA). Statistical differences in LDH release and cytokine secretion between RAW 264.7 macrophages challenged with A. fumigatus CAPA isolates and reference strains were determined by one-way multiparametric ANOVA with Dunnett’s correction using GraphPad Prism 9.0 (La Jolla, CA, USA).

### Data availability.

Assembled genomes and annotations used in this study are available via Figshare at https://figshare.com/articles/dataset/COVID-19_Associated_Pulmonary_Aspergillosis_Isolates_are_genomically_diverse_but_are_more_similar_to_each_other_in_their_responses_to_infection-relevant_stresses/20409096. Reads, assemblies, and annotations that met NCBI formatting guidelines and are very similar to those discussed here, are available through BioProject PRJNA787571. Note that for the NCBI genomes, “Sample #” is synonymous with “CAPA #.”

## References

[1] Gangneux J-P, Dannaoui E, Fekkar A, Luyt C-E, Botterel F, De Prost N, Tadié J-M, Reizine F, Houzé S, Timsit J-F, Iriart X, Riu-Poulenc B, Sendid B, Nseir S, Persat F, Wallet F, Le Pape P, Canet E, Novara A, Manai M, Cateau E, Thille AW, Brun S, Cohen Y, Alanio A, Mégarbane B, Cornet M, Terzi N, Lamhaut L, Sabourin E, Desoubeaux G, Ehrmann S, Hennequin C, Voiriot G, Nevez G, Aubron C, Letscher-Bru V, Meziani F, Blaize M, Mayaux J, Monsel A, Boquel F, Robert-Gangneux F, Le Tulzo Y, Seguin P, Guegan H, Autier B, Lesouhaitier M, Pelletier R, Belaz S, et al. 2022. Fungal infections in mechanically ventilated patients with COVID-19 during the first wave: the French multicentre MYCOVID study. Lancet Respir Med 10:180–190. doi:10.1016/S2213-2600(21)00442-2. 34843666 PMC8626095

[2] Hoenigl M, Seidel D, Sprute R, Cunha C, Oliverio M, Goldman GH, Ibrahim AS, Carvalho A. 2022. COVID-19-associated fungal infections. Nat Microbiol 7:1127–1140. doi:10.1038/s41564-022-01172-2. 35918423 PMC9362108

[3] Feys S, Almyroudi MP, Braspenning R, Lagrou K, Spriet I, Dimopoulos G, Wauters J. 2021. A visual and comprehensive review on COVID-19-associated pulmonary aspergillosis (CAPA). J Fungi (Basel) 7:1067. doi:10.3390/jof7121067. 34947049 PMC8708864

[4] Salmanton-García J, Sprute R, Stemler J, Bartoletti M, Dupont D, Valerio M, Garcia-Vidal C, Falces-Romero I, Machado M, de la Villa S, Schroeder M, Hoyo I, Hanses F, Ferreira-Paim K, Giacobbe DR, Meis JF, Gangneux J-P, Rodríguez-Guardado A, Antinori S, Sal E, Malaj X, Seidel D, Cornely OA, Koehler P, FungiScope European Confederation of Medical Mycology/The International Society for Human and Animal Mycology Working Group. 2021. COVID-19-associated pulmonary aspergillosis, March–August 2020. Emerg Infect Dis 27:1077–1086. doi:10.3201/eid2704.204895. 33539721 PMC8007287

[5] Steenwyk JL, Mead ME, de Castro PA, Valero C, Damasio A, dos Santos RAC, Labella AL, Li Y, Knowles SL, Raja HA, Oberlies NH, Zhou X, Cornely OA, Fuchs F, Koehler P, Goldman GH, Rokas A. 2021. Genomic and phenotypic analysis of COVID-19-associated pulmonary aspergillosis isolates of *Aspergillus fumigatus*. Microbiol Spectr 9:e00010-21. doi:10.1128/Spectrum.00010-21. PMC8552514 34106569

[6] Badali H, Cañete-Gibas C, McCarthy D, Patterson H, Sanders C, David MP, Mele J, Fan H, Wiederhold NP. 2022. Species distribution and antifungal susceptibilities of *Aspergillus* section *Fumigati* isolates in clinical samples from the United States. J Clin Microbiol 60:e00280-22. doi:10.1128/jcm.00280-22. 35400175 PMC9116166

[7] Nierman WC, Pain A, Anderson MJ, Wortman JR, Kim HS, Arroyo J, Berriman M, Abe K, Archer DB, Bermejo C, Bennett J, Bowyer P, Chen D, Collins M, Coulsen R, Davies R, Dyer PS, Farman M, Fedorova N, Fedorova N, Feldblyum TV, Fischer R, Fosker N, Fraser A, García JL, García MJ, Goble A, Goldman GH, Gomi K, Griffith-Jones S, Gwilliam R, Haas B, Haas H, Harris D, Horiuchi H, Huang J, Humphray S, Jiménez J, Keller N, Khouri H, Kitamoto K, Kobayashi T, Konzack S, Kulkarni R, Kumagai T, Lafon A, Latgé J-P, Li W, Lord A, Lu C, et al. 2005. Genomic sequence of the pathogenic and allergenic filamentous fungus *Aspergillus fumigatus*. Nature 438:1151–1156. doi:10.1038/nature04332. 16372009

[8] Fedorova ND, Khaldi N, Joardar VS, Maiti R, Amedeo P, Anderson MJ, Crabtree J, Silva JC, Badger JH, Albarraq A, Angiuoli S, Bussey H, Bowyer P, Cotty PJ, Dyer PS, Egan A, Galens K, Fraser-Liggett CM, Haas BJ, Inman JM, Kent R, Lemieux S, Malavazi I, Orvis J, Roemer T, Ronning CM, Sundaram JP, Sutton G, Turner G, Venter JC, White OR, Whitty BR, Youngman P, Wolfe KH, Goldman GH, Wortman JR, Jiang B, Denning DW, Nierman WC. 2008. Genomic islands in the pathogenic filamentous fungus *Aspergillus fumigatus*. PLoS Genet 4:e1000046. doi:10.1371/journal.pgen.1000046. 18404212 PMC2289846

[9] Abad A, Fernández-Molina JV, Bikandi J, Ramírez A, Margareto J, Sendino J, Hernando FL, Pontón J, Garaizar J, Rementeria A. 2010. What makes *Aspergillus fumigatus* a successful pathogen? Genes and molecules involved in invasive aspergillosis. Revista Iberoamericana de Micologia 27:155–182. doi:10.1016/j.riam.2010.10.003. 20974273

[10] Brown NA, Goldman GH. 2016. The contribution of *Aspergillus fumigatus* stress responses to virulence and antifungal resistance. J Microbiol 54:243–253. doi:10.1007/s12275-016-5510-4. 26920884

[11] Bignell E, Cairns TC, Throckmorton K, Nierman WC, Keller NP. 2016. Secondary metabolite arsenal of an opportunistic pathogenic fungus. Philos Trans R Soc B 371:20160023–20160029. doi:10.1098/rstb.2016.0023. PMC5095546 28080993

[12] Mead ME, Steenwyk JL, Silva LP, de Castro PA, Saeed N, Hillmann F, Goldman GH, Rokas A. 2021. An evolutionary genomic approach reveals both conserved and species-specific genetic elements related to human disease in closely related *Aspergillus* fungi. Genetics 218:iyab066. doi:10.1093/genetics/iyab066. 33944921 PMC8225353

[13] Fuller KK, Cramer RA, Zegans ME, Dunlap JC, Loros JJ. 2016. *Aspergillus fumigatus* photobiology illuminates the marked heterogeneity between isolates. mBio 7:e01517-16. doi:10.1128/mBio.01517-16. 27651362 PMC5030361

[14] Kowalski CH, Beattie SR, Fuller KK, McGurk EA, Tang Y-W, Hohl TM, Obar JJ, Cramer RA. 2016. Heterogeneity among isolates reveals that fitness in low oxygen correlates with *Aspergillus fumigatus* virulence. mBio 7:e01515-16. doi:10.1128/mBio.01515-16. 27651366 PMC5040115

[15] Ries LNA, Steenwyk JL, de Castro PA, de Lima PBA, Almeida F, de Assis LJ, Manfiolli AO, Takahashi-Nakaguchi A, Kusuya Y, Hagiwara D, Takahashi H, Wang X, Obar JJ, Rokas A, Goldman GH. 2019. Nutritional heterogeneity among *Aspergillus fumigatus* strains has consequences for virulence in a strain- and host-dependent manner. Front Microbiol 10:854. doi:10.3389/fmicb.2019.00854. 31105662 PMC6492530

[16] Barber AE, Sae-Ong T, Kang K, Seelbinder B, Li J, Walther G, Panagiotou G, Kurzai O. 2021. *Aspergillus fumigatus* pan-genome analysis identifies genetic variants associated with human infection. Nat Microbiol 6:1526–1536. doi:10.1038/s41564-021-00993-x. 34819642

[17] Rhodes J, Abdolrasouli A, Dunne K, Sewell TR, Zhang Y, Ballard E, Brackin AP, van Rhijn N, Chown H, Tsitsopoulou A, Posso RB, Chotirmall SH, McElvaney NG, Murphy PG, Talento AF, Renwick J, Dyer PS, Szekely A, Bowyer P, Bromley MJ, Johnson EM, Lewis White P, Warris A, Barton RC, Schelenz S, Rogers TR, Armstrong-James D, Fisher MC. 2022. Population genomics confirms acquisition of drug-resistant *Aspergillus fumigatus* infection by humans from the environment. Nat Microbiol 7:663–674. doi:10.1038/s41564-022-01091-2. 35469019 PMC9064804

[18] Horta MAC, Steenwyk JL, Mead ME, Dos Santos LHB, Zhao S, Gibbons JG, Marcet-Houben M, Gabaldón T, Rokas A, Goldman GH. 2022. Examination of genome-wide ortholog variation in clinical and environmental isolates of the fungal pathogen *Aspergillus fumigatus*. mBio 13:e0151922. doi:10.1128/mbio.01519-22. 35766381 PMC9426589

[19] Lofgren LA, Ross BS, Cramer RA, Stajich JE. 2022. The pan-genome of *Aspergillus fumigatus* provides a high-resolution view of its population structure revealing high levels of lineage-specific diversity driven by recombination. PLoS Biol 20:e3001890. doi:10.1371/journal.pbio.3001890. 36395320 PMC9714929

[20] Engel T, Verweij PE, van den Heuvel J, Wangmo D, Zhang J, Debets AJM, Snelders E. 2020. Parasexual recombination enables *Aspergillus fumigatus* to persist in cystic fibrosis. ERJ Open Res 6:e00020. doi:10.1183/23120541.00020-2020. PMC7720686 33313304

[21] Ballard E, Melchers WJG, Zoll J, Brown AJP, Verweij PE, Warris A. 2018. In-host microevolution of *Aspergillus fumigatus*: a phenotypic and genotypic analysis. Fungal Genet Biol 113:1–13. doi:10.1016/j.fgb.2018.02.003. 29477713 PMC5883321

[22] Steenwyk JL, Buida TJ, Li Y, Shen X-X, Rokas A. 2020. ClipKIT: a multiple sequence alignment trimming software for accurate phylogenomic inference. PLoS Biol 18:e3001007. doi:10.1371/journal.pbio.3001007. 33264284 PMC7735675

[23] Houbraken J, Weig M, Groß U, Meijer M, Bader O. 2016. *Aspergillus oerlinghausenensis*, a new mould species closely related to *A. fumigatus*. FEMS Microbiology Lett 363:fnv236. doi:10.1093/femsle/fnv236. 26667219

[24] Arendrup MC, Friberg N, Mares M, Kahlmeter G, Meletiadis J, Guinea J, Arendrup MC, Meletiadis J, Guinea J, Friberg N, Mares M, Kahlmeter G, Andersen CT, Arikan-Akdagli S, Barchiesi F, Chryssanthou E, Hamal P, Järv H, Klimko N, Kurzai O, Lagrou K, Lass-Flörl C, Matos T, Muehlethaler K, Rogers TR, Velegraki A, Arikan S, Subcommittee on Antifungal Susceptibility Testing (AFST) of the ESCMID European Committee for Antimicrobial Susceptibility Testing (EUCAST). 2020. How to interpret MICs of antifungal compounds according to the revised clinical breakpoints v. 10.0 European committee on antimicrobial susceptibility testing (EUCAST). Clin Microbiol Infect 26:1464–1472. doi:10.1016/j.cmi.2020.06.007. 32562861

[25] European Committee on Antimicrobial Susceptibility Testing. 2020. Breakpoint tables for interpretation of MICs for antifungal agents. European Committee for Antimicrobial Susceptibility Testing.

[26] Knoll R, Schultze JL, Schulte-Schrepping J. 2021. Monocytes and macrophages in COVID-19. Front Immunol 12:720109. doi:10.3389/fimmu.2021.720109. 34367190 PMC8335157

[27] Knox BP, Blachowicz A, Palmer JM, Romsdahl J, Huttenlocher A, Wang CCC, Keller NP, Venkateswaran K. 2016. Characterization of *Aspergillus fumigatus* isolates from air and surfaces of the International Space Station. mSphere 1:e00227-16. doi:10.1128/mSphere.00227-16. 27830189 PMC5082629

[28] Caffrey-Carr AK, Kowalski CH, Beattie SR, Blaseg NA, Upshaw CR, Thammahong A, Lust HE, Tang Y-W, Hohl TM, Cramer RA, Obar JJ. 2017. Interleukin 1α is critical for resistance against highly virulent *Aspergillus fumigatus* isolates. Infect Immun 85:e00661-17. doi:10.1128/IAI.00661-17. 28947643 PMC5695118

[29] Rosowski EE, Raffa N, Knox BP, Golenberg N, Keller NP, Huttenlocher A. 2018. Macrophages inhibit *Aspergillus fumigatus* germination and neutrophil-mediated fungal killing. PLoS Pathog 14:e1007229. doi:10.1371/journal.ppat.1007229. 30071103 PMC6091969

[30] Bertuzzi M, van Rhijn N, Krappmann S, Bowyer P, Bromley MJ, Bignell EM. 2021. On the lineage of *Aspergillus fumigatus* isolates in common laboratory use. Med Mycol 59:7–13. doi:10.1093/mmy/myaa075. 32944768 PMC7779236

[31] Dancer P, Pickard A, Potocka W, Fortune-Grant R, Earle K, Kadler KE, Bertuzzi M, Gago S. (2022). Mutual inhibition of airway epithelial responses supports viral and fungal co-pathogenesis during coinfection. bioRxiv. doi:10.1101/2022.04.13.488236.

[32] Meijer EFJ, Dofferhoff ASM, Hoiting O, Meis JF. 2021. COVID-19-associated pulmonary aspergillosis: a prospective single-center dual case series. Mycoses 64:457–464. doi:10.1111/myc.13254. 33569857 PMC7986084

[33] Kirchhoff L, Braun LM, Schmidt D, Dittmer S, Dedy J, Herbstreit F, Stauf R, Steckel NK, Buer J, Rath P, Steinmann J, Verhasselt HL. 2022. COVID-19-associated pulmonary aspergillosis in ICU patients in a German reference centre: phenotypic and molecular characterisation of *Aspergillus fumigatus* isolates. Mycoses 65:458–465. doi:10.1111/myc.13430. 35138651 PMC9115305

[34] Erami M, Hashemi SJ, Raiesi O, Fattahi M, Getso MI, Momen-Heravi M, Daie Ghazvini R, Khodavaisy S, Parviz S, Mehri N, Babaei M. 2023. COVID-19-associated pulmonary aspergillosis (CAPA) in Iranian patients admitted with severe COVID-19 pneumonia. Infection 51:223–230. doi:10.1007/s15010-022-01907-7. 36107379 PMC9476444

[35] Peláez-García de la Rasilla T, González-Jiménez I, Fernández-Arroyo A, Roldán A, Carretero-Ares JL, García-Clemente M, Telenti-Asensio M, García-Prieto E, Martínez-Suarez M, Vázquez-Valdés F, Melón-García S, Caminal-Montero L, Fernández-Simón I, Mellado E, Sánchez-Núñez ML. 2022. COVID-19 associated pulmonary aspergillosis (CAPA): hospital or home environment as a source of life-threatening *Aspergillus fumigatus* infection? JoF 8:316. doi:10.3390/jof8030316. 35330318 PMC8952274

[36] Prattes J, Wauters J, Giacobbe DR, Salmanton-García J, Maertens J, Bourgeois M, Reynders M, Rutsaert L, Van Regenmortel N, Lormans P, Feys S, Reisinger AC, Cornely OA, Lahmer T, Valerio M, Delhaes L, Jabeen K, Steinmann J, Chamula M, Bassetti M, Hatzl S, Rautemaa-Richardson R, Koehler P, Lagrou K, Hoenigl M, Debaveye Y, Miceli MH, Tudesq JJ, Paul G, Krause R, Linhofer M, Frost J, Zechner P, Kochanek M, Eller P, Jenks JD, Volpi S, Bellanger AP, White PL, Goldman GH, Bowyer P, Rokas A, Gago S, Pelosi P, Robba C, Gangneux JP, Lass-Floerl C, Machado M, Muñoz P. 2022. Risk factors and outcome of pulmonary aspergillosis in critically ill coronavirus disease 2019 patients—a multinational observational study by the European Confederation of Medical Mycology. Clin Microbiol Infect 28:580–587. doi:10.1016/j.cmi.2021.08.014. 34454093 PMC8387556

[37] Barratt RW, Johnson GB, Ogata WN. 1965. Wild-type and mutant stocks of *Aspergillus nidulans*. Genetics 52:233–246. doi:10.1093/genetics/52.1.233. 5857598 PMC1210840

[38] Käfer E. 1977. Meiotic and mitotic recombination in *Aspergillus* and its chromosomal aberrations. Adv Genet 19:33–131. doi:10.1016/S0065-2660(08)60245-X. 327767

[39] Bolger AM, Lohse M, Usadel B. 2014. Trimmomatic: a flexible trimmer for Illumina sequence data. Bioinformatics 30:2114–2120. doi:10.1093/bioinformatics/btu170. 24695404 PMC4103590

[40] Prjibelski A, Antipov D, Meleshko D, Lapidus A, Korobeynikov A. 2020. Using SPAdes De Novo Assembler. Current Protocols in Bioinformatics 70:e102. doi:10.1002/cpbi.102. 32559359

[41] Steenwyk JL, Buida TJ, Gonçalves C, Goltz DC, Morales G, Mead ME, LaBella AL, Chavez CM, Schmitz JE, Hadjifrangiskou M, Li Y, Rokas A. 2022. BioKIT: a versatile toolkit for processing and analyzing diverse types of sequence data. Genetics 221:iyac079. doi:10.1093/genetics/iyac079. 35536198 PMC9252278

[42] Stanke M, Diekhans M, Baertsch R, Haussler D. 2008. Using native and syntenically mapped cDNA alignments to improve *de novo* gene finding. Bioinformatics 24:637–644. doi:10.1093/bioinformatics/btn013. 18218656

[43] Manni M, Berkeley MR, Seppey M, Simão FA, Zdobnov EM. 2021. BUSCO update: novel and streamlined workflows along with broader and deeper phylogenetic coverage for scoring of eukaryotic, prokaryotic, and viral genomes. Mol Biol Evol 38:4647–4654. doi:10.1093/molbev/msab199. 34320186 PMC8476166

[44] Amos B, Aurrecoechea C, Barba M, Barreto A, Basenko EY, Bażant W, Belnap R, Blevins AS, Böhme U, Brestelli J, Brunk BP, Caddick M, Callan D, Campbell L, Christensen MB, Christophides GK, Crouch K, Davis K, DeBarry J, Doherty R, Duan Y, Dunn M, Falke D, Fisher S, Flicek P, Fox B, Gajria B, Giraldo-Calderón GI, Harb OS, Harper E, Hertz-Fowler C, Hickman MJ, Howington C, Hu S, Humphrey J, Iodice J, Jones A, Judkins J, Kelly SA, Kissinger JC, Kwon DK, Lamoureux K, Lawson D, Li W, Lies K, Lodha D, Long J, MacCallum RM, Maslen G, McDowell MA, et al. 2022. VEuPathDB: the eukaryotic pathogen, vector and host bioinformatics resource center. Nucleic Acids Res 50:D898–D911. doi:10.1093/nar/gkab929. 34718728 PMC8728164

[45] Altschul SF, Gish W, Miller W, Myers EW, Lipman DJ. 1990. Basic local alignment search tool. J Mol Biol 215:403–410. doi:10.1016/S0022-2836(05)80360-2. 2231712

[46] Katoh K, Misawa K, Kuma K, Miyata T. 2002. MAFFT: a novel method for rapid multiple sequence alignment based on fast Fourier transform. Nucleic Acids Res 30:3059–3066. doi:10.1093/nar/gkf436. 12136088 PMC135756

[47] Katoh K, Standley DM. 2013. MAFFT multiple sequence alignment software version 7: improvements in performance and usability. Mol Biol Evol 30:772–780. doi:10.1093/molbev/mst010. 23329690 PMC3603318

[48] Nguyen L-T, Schmidt HA, von Haeseler A, Minh BQ. 2015. IQ-TREE: a fast and effective stochastic algorithm for estimating maximum-likelihood phylogenies. Mol Biol Evol 32:268–274. doi:10.1093/molbev/msu300. 25371430 PMC4271533

[49] Letunic I, Bork P. 2021. Interactive Tree Of Life (iTOL) v5: an online tool for phylogenetic tree display and annotation. Nucleic Acids Res 49:W293–W296. doi:10.1093/nar/gkab301. 33885785 PMC8265157

[50] Steenwyk JL, Rokas A. 2021. orthofisher: a broadly applicable tool for automated gene identification and retrieval. G3 (Bethesda) 11:jkab250. doi:10.1093/g3journal/jkab250. 34544141 PMC8496211

[51] Steenwyk JL, Buida TJ, Labella AL, Li Y, Shen X-X, Rokas A. 2021. PhyKIT: a broadly applicable UNIX shell toolkit for processing and analyzing phylogenomic data. Bioinformatics 37:2325–2331. doi:10.1093/bioinformatics/btab096. 33560364 PMC8388027

[52] Jones DT, Taylor WR, Thornton JM. 1992. The rapid generation of mutation data matrices from protein sequences. Comput Appl Biosci 8:275–282. 1633570 10.1093/bioinformatics/8.3.275

[53] Hoang DT, Chernomor O, von Haeseler A, Minh BQ, Vinh LS. 2018. UFBoot2: improving the ultrafast bootstrap approximation. Mol Biol Evol 35:518–522. doi:10.1093/molbev/msx281. 29077904 PMC5850222

[54] Arendrup MC, Meletiadis J, Lagrou K, Hamal P, Guinea J, Subcommitee on Antifungal Suceptibility Testing (AFST) of the ESCMID European Committee for Antimicrobial Susceptibility Testing (EUCAST). 2020. EUCAST definitive document E.DEF 9.3.2: method for the determination of broth dilution minimum inhibitory concentrations of antifungal agents for conidia forming moulds. European Committee for Antimicrobial Susceptibility Testing.

[55] Gsaller F, Hortschansky P, Beattie SR, Klammer V, Tuppatsch K, Lechner BE, Rietzschel N, Werner ER, Vogan AA, Chung D, Mühlenhoff U, Kato M, Cramer RA, Brakhage AA, Haas H. 2014. The Janus transcription factor HapX controls fungal adaptation to both iron starvation and iron excess. EMBO J 33:2261–2276. doi:10.15252/embj.201489468. 25092765 PMC4232046

[56] Furukawa T, van Rhijn N, Fraczek M, Gsaller F, Davies E, Carr P, Gago S, Fortune-Grant R, Rahman S, Gilsenan JM, Houlder E, Kowalski CH, Raj S, Paul S, Cook P, Parker JE, Kelly S, Cramer RA, Latgé J-P, Moye-Rowley S, Bignell E, Bowyer P, Bromley MJ. 2020. The negative cofactor 2 complex is a key regulator of drug resistance in *Aspergillus fumigatus*. Nat Commun 11:427. doi:10.1038/s41467-019-14191-1. 31969561 PMC7194077

